# Hypofractionated Gamma Knife Radiosurgery for Large Brain Metastases in Surgery-Ineligible Patients: Outcomes of a Uniform 5-Fraction Regimen

**DOI:** 10.3390/cancers18091475

**Published:** 2026-05-03

**Authors:** Juhee Jeon, Yukyeng Byeon, Gung Ju Kim, Yoohyun Kwon, Suhmi Chung, Do Hee Lee, Sang Woo Song, Young Hyun Cho, Chang-Ki Hong, Seok Ho Hong, Jeong-Hoon Kim, Young-Hoon Kim

**Affiliations:** 1 Department of Neurological Surgery, Asan Medical Center, University of Ulsan College of Medicine, Seoul 05505, Republic of Korea; doctorjuhee88@gmail.com (J.J.); na1412@amc.seoul.kr (Y.B.); d190220@amc.seoul.kr (G.J.K.); uhyun06@gmail.com (Y.K.); d250116@amc.seoul.kr (S.C.); a250377@amc.seoul.kr (D.H.L.); his4u2@amc.seoul.kr (S.W.S.); yhyunc@amc.seoul.kr (Y.H.C.); changki.hong@amc.seoul.kr (C.-K.H.); hongsound@amc.seoul.kr (S.H.H.); jhkim1@amc.seoul.kr (J.-H.K.); 2 Department of Neurosurgery, Uijeongbu Eulji Medical Center, Eulji University College of Medicine, Uijeongbu-si 11759, Republic of Korea

**Keywords:** brain metastases, stereotactic radiotherapy, hypofractionated radiosurgery, Gamma Knife, surgery-ineligible, radiation necrosis

## Abstract

Background: Surgical resection remains the standard treatment for large brain metastases (LBMs), but many patients are not surgical candidates due to poor performance status or uncontrolled systemic disease. Gamma Knife-based hypofractionated stereotactic radiotherapy (GKRS) has emerged as a potential alternative; however, its clinical role in this population remains insufficiently defined. We evaluated whether a uniform daily 5-fraction GKRS provides effective and safe local treatment for surgery-ineligible LBMs. Methods: We retrospectively analyzed 100 patients with LBMs (>14 cm^3^) who underwent primary hypofractionated GKRS using a uniform daily 5-fraction schedule. Forty-six patients were male; the median age was 60 years. The median Karnofsky Performance Status (KPS) was 70 (60–100); a total of 47 patients (47%) had pre-GKRS neurological deficits. The most common primary sites were lung (41), breast (24), and kidney (14). The median tumor volume was 22.0 cm^3^ (14–70 cm^3^), and the marginal dose was 35.2 Gy (50% isodose line) in 5 fractions. The primary endpoints included local tumor control (LTC), intracranial progression-free survival (PFS), and overall survival (OS). Radiation necrosis (RN) was assessed as a key safety outcome. Results: At a median follow-up of 18 months, the overall LTC rate was 74%, with 1-, 2-, and 3-year rates of 73%, 65%, and 60%, respectively. Median PFS and OS were 7.5 and 16.3 months. Higher pre-treatment KPS and absence of neurological deficits were independently associated with improved OS (*p* = 0.003 and 0.025, respectively). RN occurred in 16% of patients, with 9% developing symptoms; all symptomatic cases were effectively managed with corticosteroids or bevacizumab. Most tumors demonstrated substantial volumetric reduction, with a median decrease of 80% and 30% achieving near-complete response (>95%). Conclusions: A uniform daily 5-fraction hypofractionated GKRS provides effective local control with acceptable toxicity in patients with LBMs. These findings support its role as a feasible local treatment option in selected patients who are not candidates for surgery. Integration with systemic therapies and prospective validation are warranted to refine patient selection and optimize outcomes.

## Simple Summary

Large brain metastases are commonly treated with surgical resection; however, many patients are not eligible for surgery due to poor clinical condition or advanced systemic disease. In this study, we evaluated a five-fraction Gamma Knife-based hypofractionated stereotactic radiosurgery regimen as a non-surgical treatment option in 100 patients with large brain metastases. This approach achieved good local tumor control with substantial tumor shrinkage in most cases and acceptable toxicity. Although radiation necrosis occurred in a minority of patients, it was generally manageable with medical treatment. Overall, our findings suggest that fractionated Gamma Knife radiosurgery is a safe and effective non-surgical local treatment option in appropriately selected patients and may help expand treatment options for this challenging population.

## 1. Introduction

Brain metastases are the most common malignant intracranial tumors. Despite this prevalence, optimal treatment remains controversial [1]. Randomized trials have shown that, in patients with a single brain metastasis, good performance status, and limited extracranial disease, adjuvant radiotherapy after resection improves overall survival (OS) and reduces recurrence [2,3,4]. More recently, stereotactic radiosurgery (SRS) has been recommended as an effective primary treatment for patients not suitable for surgery [5,6].

According to National Comprehensive Cancer Network guidelines, resection is recommended for patients with mass effect or neurological symptoms, newly diagnosed or stable systemic disease with reasonable systemic options, or when biopsy confirmation is required [7]. SRS is also preferred for the surgical cavity and as upfront therapy for small tumor volumes (<2 cm^3^) [7]. However, a substantial proportion of patients with large brain metastases (LBMs) are not suitable candidates for surgical resection because of poor performance status, comorbidities, or advanced systemic disease, creating a critical need for effective non-surgical treatment strategies.

Although SRS is effective for small- to medium-sized metastases, treating LBMs remains challenging because of adverse effects including radiation necrosis (RN) and neurological decline [5,6,8]. The Gamma Knife Icon enables frameless hypofractionated SRS; consequently, Gamma Knife radiosurgery (GKRS) is increasingly used for LBMs. However, optimal fraction number and dose for hypofractionated SRS have not been established, and evidence regarding effectiveness, safety, and indications remains limited [5].

Therefore, we evaluated the clinical outcomes and safety of a uniform daily 5-fraction GKRS regimen for LBMs (>14 cm^3^), with a particular focus on its role as a feasible local treatment option in patients who are not candidates for surgical resection, and aimed to identify clinical parameters associated with favorable outcomes.

## 2. Methods

### 2.1. Study Cohort

Between 2017 and 2022, 2589 patients with 9211 brain metastases underwent GKRS at our institution. A total of 226 GKRS procedures (2%) targeted LBMs (>14 cm^3^). Of the 226 patients, 7 (3%) with single-fraction, 2 (1%) with 2-fraction, 40 (18%) with 3-fraction, 14 (6%) with 4-fraction, and 1 (0%) with 10-fraction GKRS were excluded. Of 162 patients (72%) who received 5-fraction GKRS for LBMs, we excluded 36 (22%) without available radiological follow-up and 26 (16%) who did not undergo primary GKRS. Subsequent review of the 36 patients excluded for unavailable post-treatment MRI showed that most had rapidly progressive systemic disease, with early death or transfer of care to outside institutions. Ultimately, 100 patients were enrolled.

Patients were selected for GKRS because they were considered unsuitable for surgical resection based on multidisciplinary clinical judgment, including poor performance status, medical comorbidities, advanced systemic disease, multifocal intracranial disease, or lesions located in surgically challenging or functionally eloquent brain regions. Multiple factors often coexisted. The study adhered to the ethical principles of the Declaration of Helsinki, and institutional review board approval was obtained.

The median age at GKRS was 60 years (range, 32–91 years), and 54 patients (54%) were female. The pre-GKRS Karnofsky Performance Status (KPS) was 60 in 10 patients (10%), 70 in 43 (43%), 80 in 33 (33%), 90 in 12 (12%), and 100 in 2 (2%). Forty-seven patients (47%) had pre-GKRS neurological deficits. The most common primary cancer was non-small cell lung cancer (NSCLC) (41%), followed by breast (24%), genitourinary (16%), gastrointestinal (7%), and small cell lung cancer (4%). Forty-three patients (43%) had extracranial metastases. Four patients (4%) received whole brain radiotherapy (WBRT) before GKRS. The median follow-up duration was 18 months (range, 3–72 months). Data regarding systemic therapies administered before or after GKRS, including targeted therapy/TKIs and immunotherapy, were additionally reviewed from medical records. Demographic and clinical characteristics are summarized in Table 1.

### 2.2. Radiosurgical Protocol

Patients underwent GKRS when surgical resection was not favored after multidisciplinary clinical evaluation because of medical, oncologic, anatomical, or symptom-related considerations. All radiosurgery procedures were performed with a Leksell Gamma Knife Icon system (Elekta, Stockholm, Sweden). A frameless mask was placed on each patient’s face. Patients underwent gadolinium-enhanced magnetic resonance imaging (MRI) with T1- and T2-weighted sequences acquired with 1.0 mm slices. Leksell GammaPlan software (version 10.2.1; Elekta, Stockholm, Sweden) was used to calculate point doses and generate dose–distance curves [9].

The number of brain metastases was 1 in 33 patients (33%), 2–5 in 48 (48%), and >5 in 19 (19%). The median tumor volume was 22.0 cm^3^ (range, 14.1–69.5 cm^3^). Tumors most commonly involved the frontal lobe (27%), followed by the parietal lobe (20%), cerebellum (18%), occipital lobe (16%), and temporal lobe (13%). All patients received 5 daily fractions. At our institution, a total dose of 35.2 Gy (approximately 7 Gy per fraction) was selected based on a biologically effective dose (BED) model. Using the linear–quadratic formula and assuming an α/β ratio of 10 for metastatic tumor tissue, this regimen corresponds to a BED10 of approximately 60 Gy, which was considered sufficient for tumor control while allowing fractionation to reduce the risk of radiation necrosis in large lesions [5,10,11]. The median marginal dose was 35.2 Gy (range, 24.9–41.5 Gy) at the 50% isodose line; a total of 71 patients (71%) received 35.2 Gy. Radiosurgical parameters are summarized in Table 1 and Table 2.

Gross tumor volume (GTV) was defined as the contrast-enhancing lesion volume on 1.0 mm axial contrast-enhanced T1-weighted MRI [9]. The clinical target volume was equal to the GTV [12]. The planning target volume (PTV) was created by expanding the contrast-enhancing GTV by 1.0 mm to account for setup uncertainty in frameless mask-based fractionated treatment [12,13]. For dosimetric analysis, the irradiated normal brain volume was defined as the whole brain volume receiving the specified dose level after subtraction of the GTV (brain-GTV). BV25 and BV30 represented the normal brain volumes receiving ≥25 Gy and ≥30 Gy, respectively.

### 2.3. Radiological and Clinical Evaluation

After GKRS, clinical assessments and follow-up MRI were performed every 3 months until last follow-up or death. Lesion volume was measured by outlining the GTV on each thin-section slice of the contrast-enhanced T1-weighted axial images, calculating the area, and summing across slices at each visit. Baseline tumor volume was the volume measured on MRI at the time of GKRS. Percentage change was calculated relative to this baseline on each follow-up MRI. Tumor reduction was defined as a >20% decrease in volume; stabilization as a change within ±20%; and progression was defined as >20% increase in lesion volume after exclusion of radiation necrosis based on clinical course and multimodal imaging assessment. These retrospective volumetric criteria were selected to reflect clinically meaningful radiographic change and were broadly aligned with the principles of the Response Assessment in Neuro-Oncology Brain Metastases (RANO-BM) framework for standardized response evaluation [14]. Best response was defined as the greatest volume reduction of the GKRS-treated lesion; its timing and magnitude were recorded.

RN was diagnosed on clinical and imaging grounds after excluding disease progression, or by histopathology when resection was performed. RN was defined as an enlarging enhancing lesion with geographic intratumoral necrosis and increasing peritumoral edema that subsequently responded to steroids without additional therapy [15,16,17]. When differentiation was challenging, experienced radiologists were consulted, and adjunct tests—including perfusion MRI, diffusion MRI, MR spectroscopy, and positron emission tomography—were obtained when progression and RN could not be reliably distinguished on conventional MRI. Initial management was steroid administration; bevacizumab was used when neurological symptoms progressed despite steroids or when adverse effects were severe. Comprehensive clinical and neurological examinations were performed at each follow-up visit.

### 2.4. Statistical Analysis

Local tumor control (LTC), intracranial progression-free survival (PFS), and OS were the primary endpoints. LTC was defined as no progression of GKRS-treated LBMs, whereas distant progression was defined as the development of new intracranial metastases outside the treated lesion. Intracranial PFS was defined as the composite of local and distant progression. OS was defined as the time from GKRS to death.

Cumulative rates of LTC, intracranial PFS, and OS were estimated using the Kaplan–Meier method and compared using the log-rank test. Variables with clinical relevance or *p* < 0.10 on univariable analysis were entered into multivariable Cox proportional hazards regression models. Hazard ratios (HRs) with 95% confidence intervals (CIs) were calculated. A two-sided *p*-value < 0.05 was considered statistically significant. All analyses were conducted using SPSS Statistics for Windows, version 22.0 (IBM Corp., Armonk, NY, USA).

## 3. Results

### 3.1. Local Tumor Control

**Figure 1:**
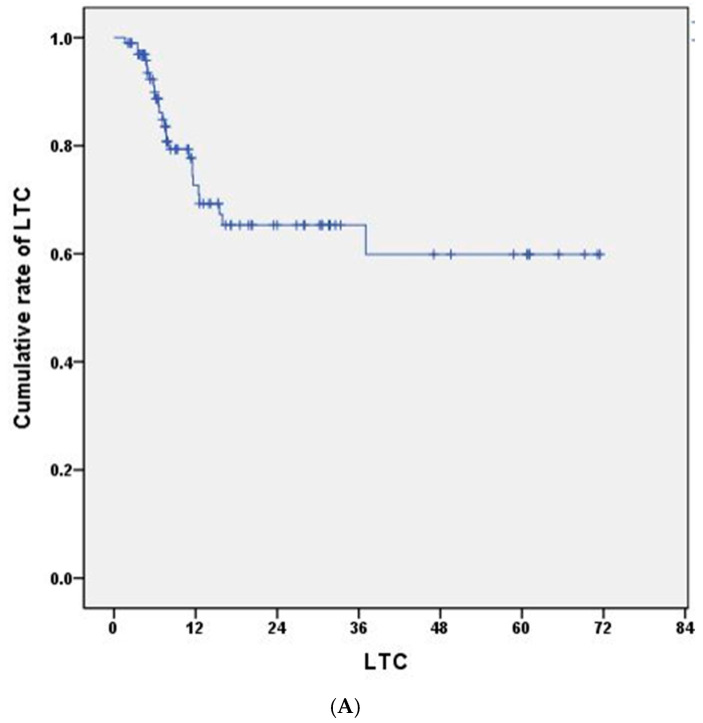

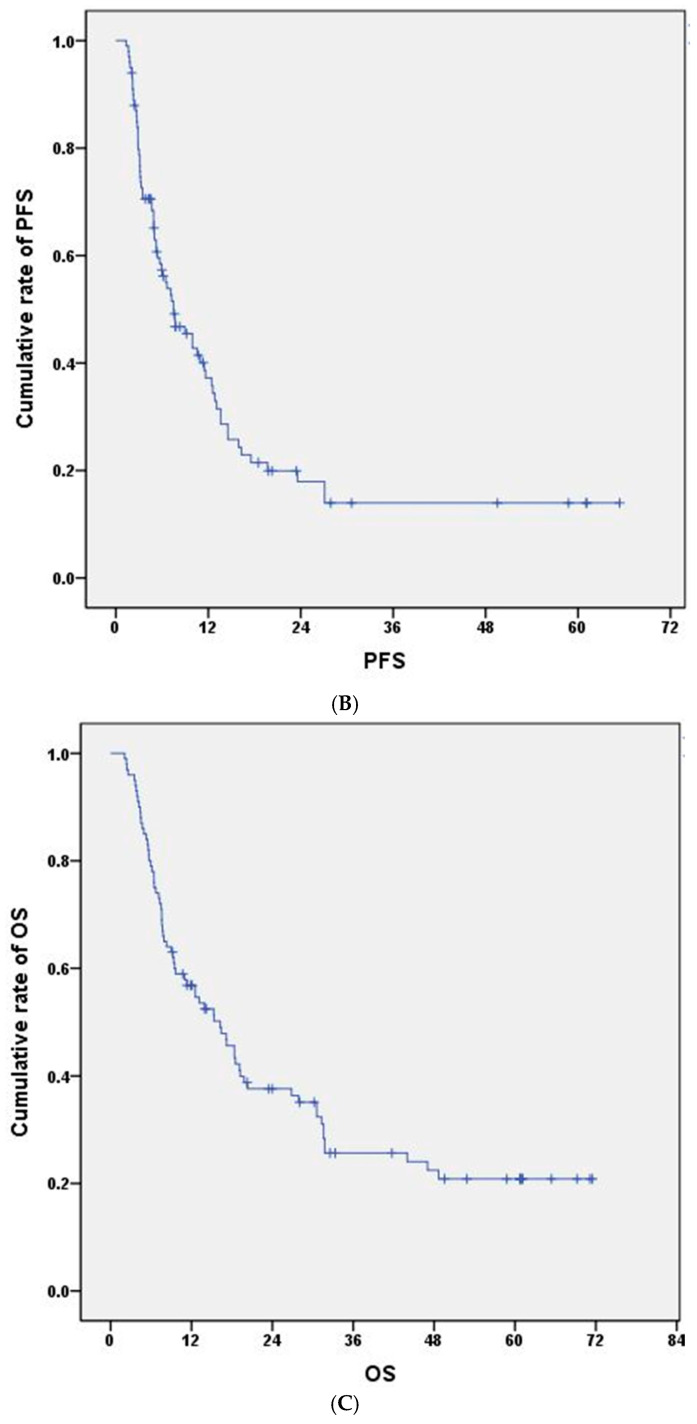
Kaplan–Meier survival curves of local tumor control (LTC) (**A**), intracranial progression-free survival (PFS) (**B**), and overall survival (OS) (**C**) of the 100 patients who underwent 5-fractionated Gamma Knife radiosurgery for large brain metastases.

During follow-up, GKRS-treated LBMs were classified as local progression after exclusion of RN through clinicoradiographic review and adjunct advanced imaging when required; overall LTC was 74%. Cumulative 1-, 2-, and 3-year LTC rates were 72.7% (95% CI, 62.3–83.1%), 65.3% (95% CI, 53.5–77.1%), and 59.9% (95% CI, 44.9–74.9%), respectively (Figure 1A). The median time from GKRS to best response was 4 months (range, 1–36 months), and 86 patients (86%) achieved best response within 1 year. Median volume reduction was 80% (range, 22–100%); a total of 30 patients (30%) had 95% reduction. Patients with NSCLC demonstrated higher LTC than other histologies, although this finding may partly reflect the influence of contemporary systemic therapies (*p* = 0.030). No significant associations were observed with age, sex, KPS, tumor location, tumor volume, or marginal dose (*p* = 0.690, 0.440, 0.872, 0.897, 0.909, and 0.645, respectively). Additional review of systemic treatments showed that 19 patients received targeted therapy or TKI-based treatment, 13 received immunotherapy, and 29 received either targeted therapy or immunotherapy during the peri-GKRS period. Among patients with NSCLC, 10 received targeted therapy and 10 received immunotherapy. Use of targeted therapy or immunotherapy was not significantly associated with local failure in the overall cohort.

### 3.2. Local and Distant Intracranial Progression

Local progression occurred in 26 patients (26%) during follow-up. In contrast, intracranial progression events were frequently driven by the development of new distant brain metastases rather than failure of the treated lesion. During follow-up duration, the median intracranial PFS was 7.5 months (95% CI, 4.6–10.4). The cumulative 1-, 2-, and 3-year intracranial PFS rates were 37.2% (95% CI, 26.6–47.8), 17.9% (95% CI, 8.7–27.1), and 14.0% (95% CI, 5.4–22.6), respectively (Figure 1B).

These findings indicate that, despite relatively favorable LTC, overall intracranial disease control was primarily limited by distant brain failure rather than local progression. Local failure status was statistically associated with intracranial PFS (*p* = 0.008); however, because local progression constitutes one component of the composite PFS endpoint, this association should be interpreted cautiously. Clinically, intracranial progression events were more commonly driven by distant brain failure than by local recurrence. However, other factors—including primary cancer type, sex, KPS, pre-GKRS neurologic deficit, pre-GKRS neurologic symptom, tumor volume, marginal dose, and RN—showed no significant association (*p* = 0.801, 0.678, 0.405, 0.610, 0.754, 0.954, 0.110, and 0.126, respectively).

### 3.3. Overall Survival

The median OS was 16.3 months (95% CI, 11.1–21.5). The cumulative 1-, 2-, and 3-year OS rates were 56.9% (95% CI, 46.9–66.9), 37.6% (95% CI, 27.6–47.6), and 25.7% (95% CI, 16.1–35.3), respectively (Figure 1C). During follow-up, 52 patients (52%) died. Of these, 27 (27%) deaths were due to progression of the primary cancer or extracranial metastases; a total of 25 (25%) deaths were attributed to persistent or recurrent brain metastases or new intracranial lesions despite GKRS.

On multivariable Cox analysis, pre-GKRS KPS > 70 (HR 0.356, 95% CI 0.168–0.756, *p* = 0.003) and absence of neurological deficits (HR 0.450, 95% CI 0.220–0.920, *p* = 0.025) were independently associated with improved OS. In contrast, patient age, sex, and primary cancer type were not significantly associated with OS (*p* = 0.203, 0.755, and 0.525, respectively). Furthermore, OS did not differ by tumor size or LTC (*p* = 0.319 and 0.846, respectively).

### 3.4. Radiation Necrosis

RN was identified in 16 (16%) patients based on follow-up imaging and outpatient clinical assessments. The median time to diagnosis on follow-up MRI was 8.5 months (range, 1.6–29.8 months) from GKRS. Among the four patients who had received prior WBRT, RN developed in two (50%). By contrast, RN occurred in 14 of 96 patients (15%) without prior WBRT. Prior WBRT was not significantly associated with RN (*p* = 0.180).

Of the 16 patients, 7 (7%) remained asymptomatic and 9 (9%) developed neurological symptoms. Based on the CTCAE (Common Terminology Criteria for Adverse Events) central nervous system necrosis grading scale, five patients (5%) had grade 2 (moderate) symptoms, and four (4%) had grade 3 symptoms, requiring hospitalization and medical intervention [17]. Neurological symptoms comprised mild headache in seven patients, nausea and vomiting in two, and lateralizing signs (partial seizures, cerebellar ataxia, or motor weakness) in four, depending on lesion location. Of the nine symptomatic patients, five had complete resolution with steroids alone, with marked reduction in peritumoral edema on follow-up MRI. In the remaining four patients with grade 3 symptoms, symptoms improved markedly after bevacizumab treatment.

Additional dosimetric analysis demonstrated that RN was more strongly associated with irradiated normal brain volume than with tumor volume alone. The median BV25 was 24.9 cm^3^ in the overall cohort, including 36.1 cm^3^ in patients who developed RN and 24.4 cm^3^ in those without RN. Likewise, the median BV30 was 13.0 cm^3^ overall, including 20.2 cm^3^ in the RN group and 12.6 cm^3^ in the non-RN group. Using a clinically relevant threshold of 20 cm^3^, patients with BV30 > 20 cm^3^ had a substantially higher incidence of RN than those with BV30 ≤ 20 cm^3^ (39.1% vs. 9.1%, *p* = 0.002). These findings suggest that limiting high-dose exposure to normal brain tissue may reduce the risk of RN after 5-fraction GKRS for large brain metastases.

### 3.5. Illustrative Cases

**Figure 2:**
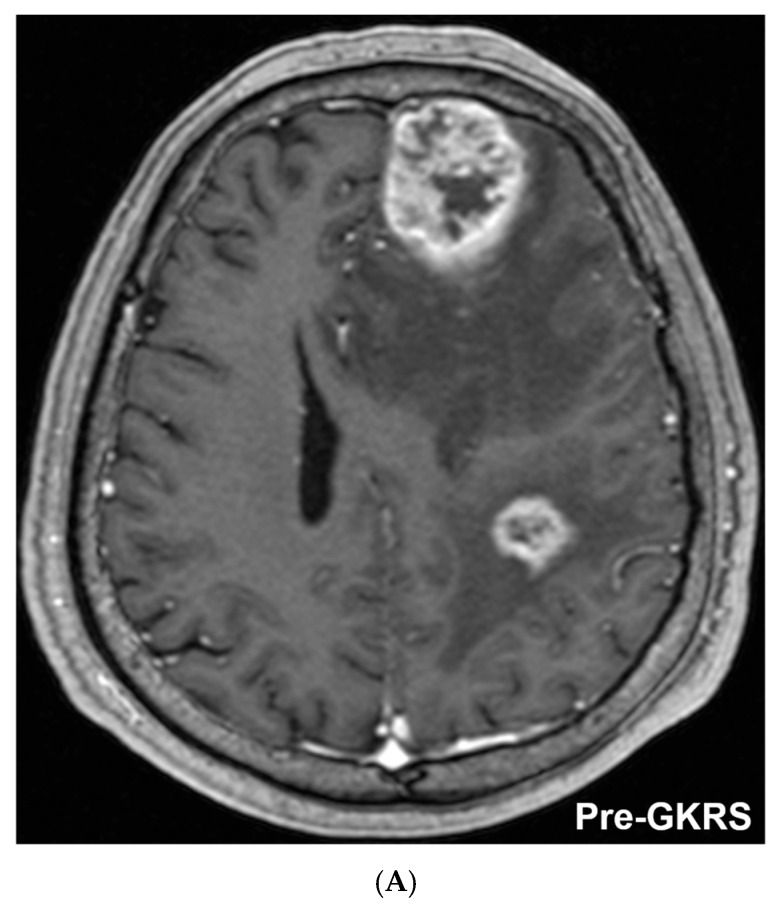

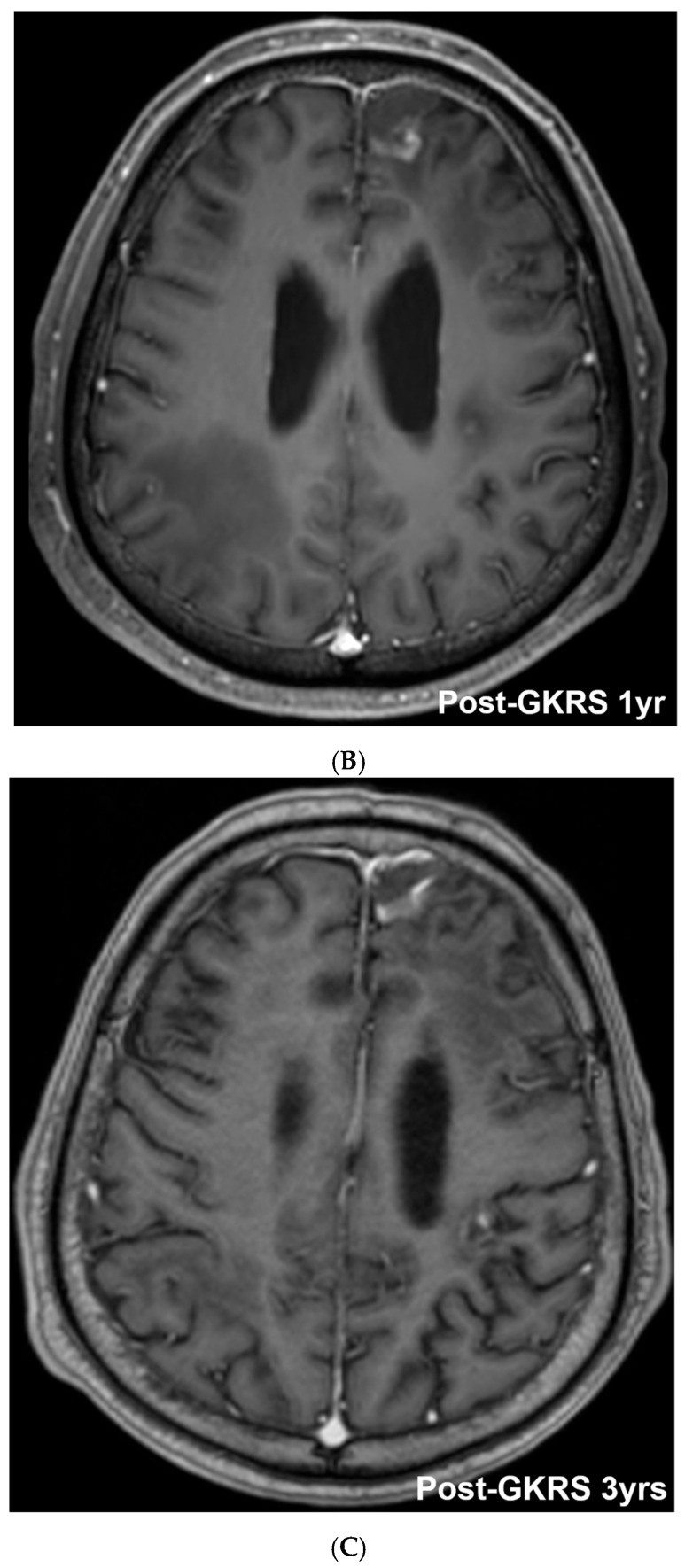
Illustrative case of a 61-year-old man with non-small cell lung cancer (squamous cell carcinoma) presenting with headache and right-sided weakness. (**A**) Pretreatment T1-weighted axial MRI demonstrating a 3.5 cm left frontal large brain metastasis (tumor volume, 23.8 cm^3^). Three additional smaller brain metastases were also identified. The left frontal lesion was treated with 5-fraction Gamma Knife radiosurgery (marginal dose, 35.2 Gy), while the smaller lesions were treated with single-fraction GKRS. (**B**) MRI obtained 1 year after treatment showing marked regression of the left frontal lesion. (**C**) Follow-up MRI 3 years after treatment demonstrating sustained local control without recurrence. The residual lesion volume was 2.0 cm^3^, corresponding to a 92% reduction from baseline.

The following cases illustrate the spectrum of responses, from durable tumor control to resolution of symptomatic RN with bevacizumab. A 61-year-old man with non-small cell lung cancer (squamous cell carcinoma) was receiving systemic chemotherapy when he presented with headache and right-sided weakness. Brain MRI revealed a 3.5 cm large brain metastasis in the left frontal lobe, along with three additional smaller metastatic lesions. The left frontal LBM had a tumor volume of 23.8 cm^3^ and was treated with 5-fraction GKRS to a marginal dose of 35.2 Gy (Figure 2A). The three smaller lesions were treated with single-fraction GKRS. At 1 year after treatment, the left frontal lesion had markedly regressed (Figure 2B), with clinical improvement of neurological symptoms. The lesion remained locally controlled without recurrence through 3 years after GKRS (Figure 2C). At the 3-year follow-up MRI, the residual tumor volume was 2.0 cm^3^, representing a 92% reduction from baseline.

**Figure 3:**
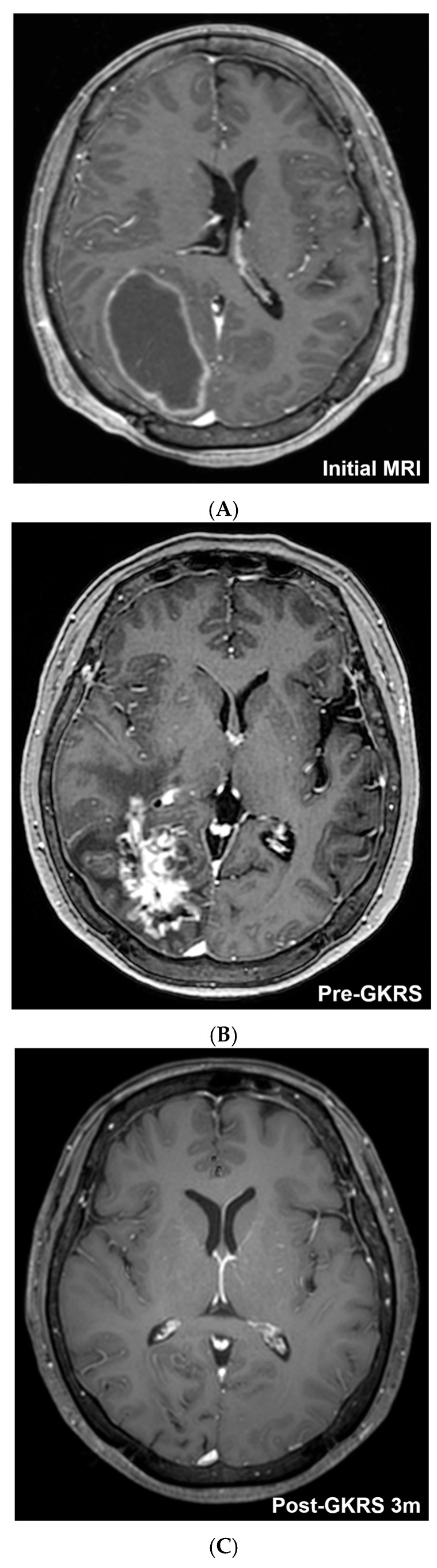

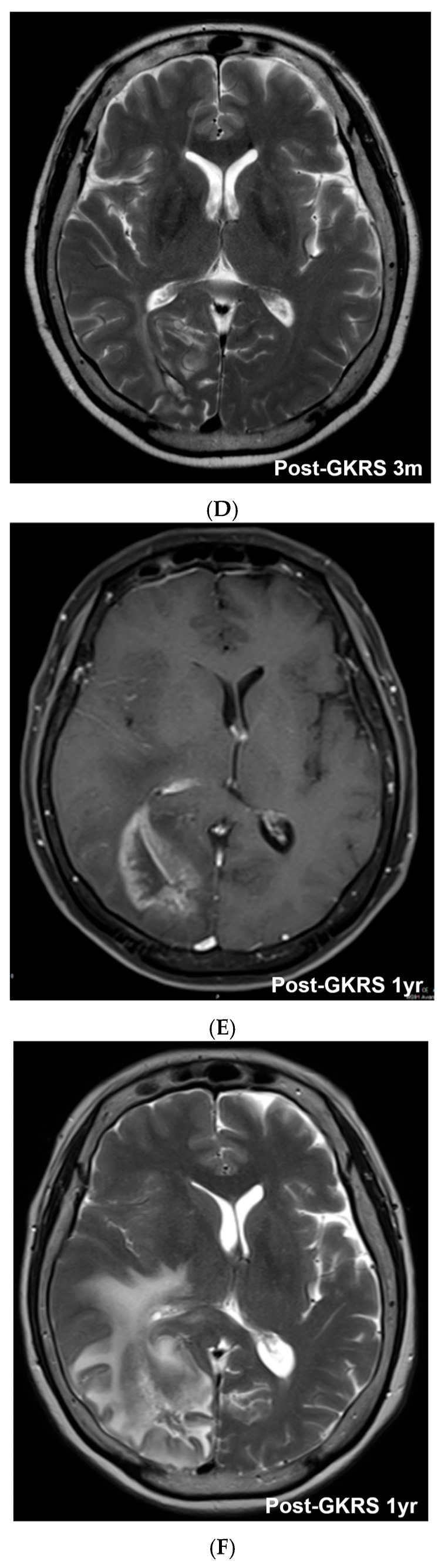

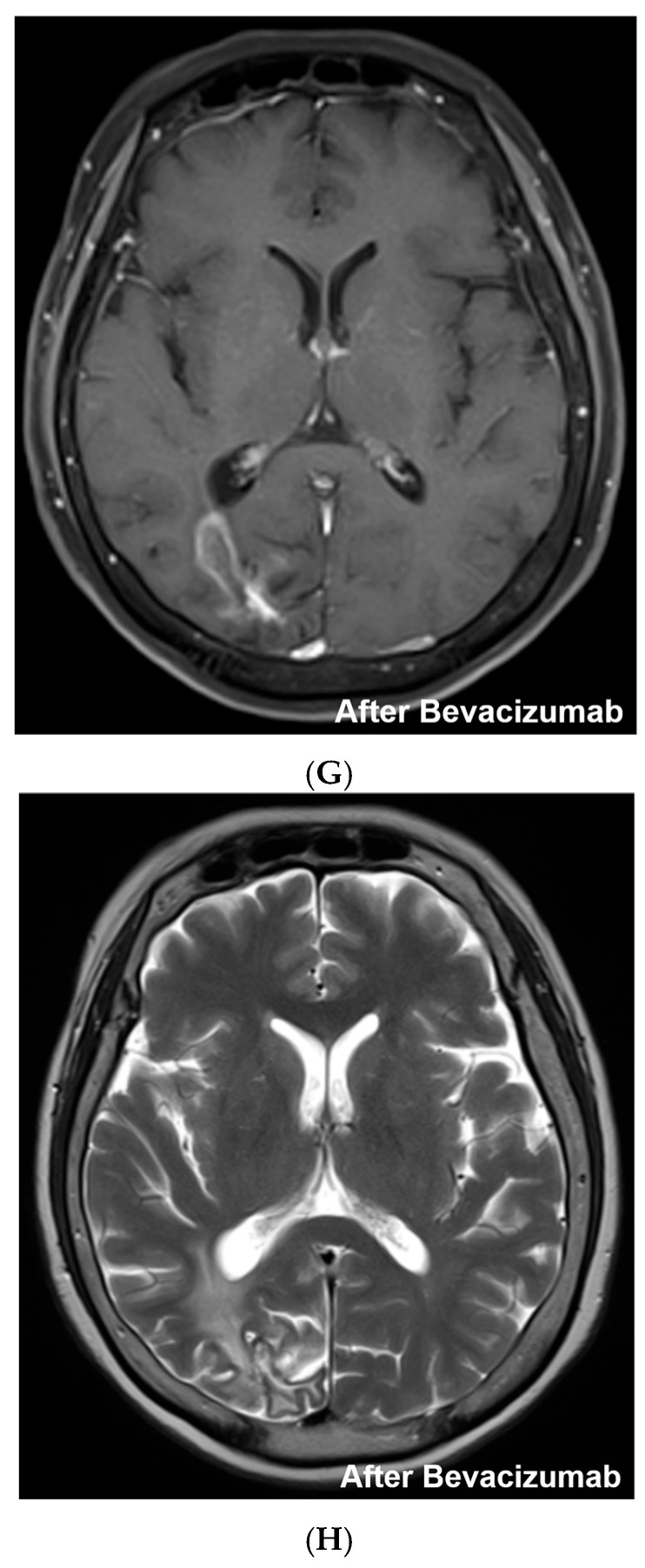
Illustrative case of a 44-year-old woman with metastatic endometrial carcinoma. (**A**) Axial T1-weighted gadolinium-enhanced MRI demonstrating a right occipital metastasis measuring approximately 67 cm^3^ with a large cystic component. (**B**) An Ommaya reservoir was inserted for cyst drainage prior to radiosurgery, reducing the lesion volume to approximately 35 cm^3^. (**C**,**D**) Three months after 5-fraction Gamma Knife radiosurgery (marginal dose, 35.2 Gy at the 50% isodose line), the lesion had markedly regressed with near-complete resolution. (**E**,**F**) Nine months later, the patient presented with headache and left hemiparesis; MRI demonstrated severe peritumoral edema with midline shift, consistent with radiation necrosis. (**G**,**H**) After bevacizumab therapy, both neurological symptoms and edema markedly improved.

A 44-year-old woman with a history of surgery for endometrial cancer, who was undergoing chemotherapy, presented with left-sided hemiparesis. T1-weighted contrast-enhanced scans (Figure 3A) revealed a right occipital brain metastasis measuring approximately 67 cm^3^ in volume, with a substantial internal cystic component. An Ommaya reservoir was placed for cyst drainage, resulting in a reduction of tumor volume to 35 cm^3^ prior to radiosurgery (Figure 3B). This approach is consistent with prior reports supporting cyst aspiration followed by fractionated Gamma Knife radiosurgery for large cystic brain metastases [18]. Subsequently, 5-fraction GKRS was performed with a marginal dose of 35.2 Gy. Three months after GKRS, the tumor had nearly resolved (Figure 3C,D). Nine months later, the patient presented to the emergency department with headache and left-sided hemiparesis. MRI revealed severe peritumoral edema with midline shift, consistent with RN (Figure 3E,F). After bevacizumab, both neurological symptoms and edema improved markedly (Figure 3G,H).

## 4. Discussion

### 4.1. Single-Fraction GKRS for LBMs

Surgical resection is the primary treatment for LBMs when lesions are accessible and performance status is favorable. When surgery is precluded by poor performance status, uncontrolled primary disease, or progressive systemic disease, SRS provides effective local control [19,20,21,22,23,24,25,26]. In this context, defining the role of radiosurgical approaches as alternatives to surgery in non-surgical candidates is of particular clinical importance. LBMs challenge radiosurgical management because safe, effective single-fraction dosing is limited [27]. In the RTOG (Radiation Therapy Oncology Group) 9005 trial, the maximum tolerated single-fraction dose for 3.1–4.0 cm lesions was 15 Gy; further escalation increased central nervous system toxicity without improving tumor control, yielding a 1-year LTC of approximately 49% [28]. Subsequent single-fraction dose-escalation studies similarly increased toxicity without improving control, producing suboptimal outcomes. For example, Vogelbaum et al. delivered single-fraction GKRS at 15 Gy and 18 Gy to tumors with median diameters of 3.3 cm (range, 2.9–4.5 cm) and 2.4 cm (range, 2.0–3.0 cm), respectively; the 1-year LTC rates were 45% and 49%, respectively [29]. These findings underscore the limitations of single-fraction SRS for LBMs.

### 4.2. Hypofractionated GKRS for LBMs

Constraints of single-fraction SRS have spurred interest in hypofractionated SRS, which distributes the total dose across multiple fractions, enabling higher biologically effective doses with less toxicity. Biologically, fractionation improves the therapeutic ratio via reoxygenation, redistribution into radiosensitive cell cycle phases, and repair of sublethal damage in normal tissue [29,30,31,32,33,34]. Gamma Knife Icon–based hypofractionated SRS combines the dosimetric precision of the GKRS platform with the radiobiological benefits of fractionation for brain metastases. Multiple series reported improved LTC and reduced toxicity versus single-fraction SRS [35,36,37,38,39]. Importantly, this concept is also supported by broader linac-based fractionated stereotactic radiotherapy literature. In an international meta-analysis of 24 studies, Lehrer et al. demonstrated that multifraction stereotactic radiosurgery for large brain metastases achieved favorable local control with reduced radionecrosis risk compared with single-fraction treatment [40]. Although prior studies have included heterogeneous fractionation schedules, tumor volumes, and patient populations, the cumulative evidence across treatment platforms supports hypofractionation as an established strategy for large brain metastases.

### 4.3. Optimal Radiosurgical Protocol for LBMs

Accordingly, we retrospectively analyzed a cohort of patients with LBMs using a uniform protocol of five daily GKRS fractions. By standardizing fractionation and restricting inclusion to clearly large tumors, we minimized confounding from dose and schedule heterogeneity. Importantly, our cohort represents a real-world population of patients who were not suitable for surgical resection because of medical frailty, systemic disease burden, multifocal disease, or lesions located in surgically high-risk anatomical regions. This reflects common multidisciplinary decision-making in contemporary practice, where treatment selection is frequently multifactorial rather than binary. Hypofractionated GKRS may be particularly valuable for large metastases in eloquent cortex, deep structures, or the posterior fossa, where surgical morbidity may be increased.

The median tumor volume in our study was 22 cm^3^ (range, 14.1–69.5 cm^3^), which is larger than that in many prior hypofractionated radiosurgery series. Treatment was delivered exclusively to LBMs using a uniform fractionation schedule. The observed LTC rates in this cohort suggest that a standardized 5-fraction regimen can achieve meaningful local disease control even in very large lesions, supporting its role as a practical treatment strategy in non-surgical candidates. This favorable outcome may partly relate to delivery of a BED10 near 60 Gy, a biologically effective dose commonly considered sufficient for metastatic tumor control while maintaining the safety advantages of fractionation [10,11].

### 4.4. Management of Radiation Necrosis

RN is the most concerning complication of radiosurgery for LBMs and is dose-limiting. Our additional analysis suggests that RN risk was more closely related to irradiated normal brain volume, particularly BV30, than to tumor size itself. This finding is consistent with prior reports emphasizing dose–volume constraints for normal brain tissue in hypofractionated treatment. Its incidence correlates with lesion size, dose per fraction, and prescription dose [28,29,41,42,43,44,45]. Single-fraction SRS for large lesions yields RN up to 20%, constraining dose escalation despite suboptimal tumor control [11,29,46,47]. Hypofractionation reduces this risk by permitting normal brain repair between fractions.

In our cohort, RN occurred in 16% of cases; only 9% were symptomatic, rates comparable with other hypofractionated GKRS series (Table 3) [5,11,48,49,50]. All symptomatic cases showed clinical and radiological improvement with corticosteroids alone or with adjunct bevacizumab. These findings suggest that, in the context of hypofractionated treatment for large lesions, RN can be effectively managed, further supporting the feasibility of this approach in patients who are not candidates for surgery.

### 4.5. Survival Impact of Hypofractionated GKRS for LBMs

OS in patients with LBMs is influenced by systemic disease burden, extracranial progression, and performance status. In our study, OS was significantly higher among patients with KPS > 70 and no neurological deficits before GKRS. Notably, while LTC was favorable, intracranial progression was frequently driven by the development of new distant metastases rather than failure of the treated lesion. This finding highlights that, although hypofractionated GKRS provides effective local treatment, overall intracranial disease control remains dependent on systemic disease status, underscoring the importance of integrating radiosurgery with contemporary systemic therapies. In our cohort, a substantial minority of patients received targeted therapy or immunotherapy, reflecting current multidisciplinary practice. Although these treatments were not significantly associated with local failure in exploratory analyses, heterogeneity in treatment timing and sequencing limits definitive interpretation of their impact. In addition, treatment outcomes may vary according to primary tumor histology. Because the majority of patients in our cohort had lung, breast, or renal cell carcinoma, the present results may not be directly generalizable to other tumor types with different biological behavior.

### 4.6. Limitations

This study had several limitations. The retrospective, single-center design limits generalizability. Retrospective analyses are vulnerable to selection bias and unmeasured confounding. In addition, 36 patients were excluded because post-treatment MRI follow-up was unavailable. Subsequent review demonstrated that 32 of these patients died of systemic disease progression, including 26 within 6 months after GKRS, while others were transferred or lacked accessible external imaging records. This may have introduced survivorship and selection bias, and limited survival in this subgroup may have reduced opportunities to detect local progression or late radiation necrosis.

Radiographic response was also assessed retrospectively using volumetric criteria rather than prospectively applying the full Response Assessment in Neuro-Oncology Brain Metastases (RANO-BM) framework, which incorporates bidimensional measurements and clinical factors. Variation in systemic therapies and follow-up protocols may also have influenced treatment outcomes. In particular, the retrospective nature of this study precluded standardized application of modern response criteria such as iRANO for patients receiving immunotherapy [51].

Furthermore, the lack of randomization precludes definitive conclusions about comparative effectiveness. Finally, given the absence of a surgical comparator group, this approach should not be interpreted as equivalent to surgical resection. Additionally, caution is warranted when extrapolating these findings to more radioresistant or hemorrhage-prone histologies, such as melanoma, which may demonstrate lower local control rates and higher risks of intratumoral hemorrhage in the setting of large brain metastases.

## 5. Conclusions

Daily 5-fraction GKRS is a safe and effective treatment for LBMs, achieving robust local control with acceptable toxicity. In patients who are not candidates for surgical resection, this approach represents a feasible local treatment option for selected patients. However, as intracranial progression is frequently driven by distant brain metastases, optimal outcomes will require integration with effective systemic therapies. Prospective multicenter studies are warranted to validate these findings and refine patient selection.

## Figures and Tables

**Table 1:** Baseline clinical and radiological characteristics of the study cohorts (*n* = 100).

Characteristic	Quantity
Sex	
Male	46 (46%)
Female	54 (54%)
Median age at GKRS, years	60 (32–91)
Median pre-GKRS KPS	70 (60–100)
60–70	53 (53%)
80–100	47 (47%)
Pre-GKRS neurological deficits	47 (47%)
Primary cancer origin	
Lung (NSCLC)	41 (41%)
Lung (SCLC)	4 (4%)
Breast	24 (24%)
Genitourinary	16 (16%)
Gastrointestinal	7 (7%)
Other *	5 (5%)
Metastasis of unknown primary	3 (3%)
Extracranial metastases	43 (43%)
Prior WBRT	4 (4%)
Prior GKRS for other brain metastases	9 (9%)
Number of brain metastases	
1	33 (33%)
2–5	48 (48%)
>5	19 (19%)
Location of large brain metastases	
Frontal lobe	27 (27%)
Parietal lobe	20 (20%)
Temporal lobe	13 (13%)
Occipital lobe	16 (16%)
Cerebellum	18 (18%)
Basal ganglia, thalamus, or insula	3 (3%)
Intraventricular	3 (3%)
Median follow-up, months	18 (3–72)

Abbreviations: GKRS = Gamma Knife radiosurgery; KPS = Karnofsky Performance Status; NSCLC = non-small cell lung cancer; SCLC = small cell lung cancer; WBRT = whole brain radiotherapy. * Other included thyroid (*n* = 2), skin (*n* = 1), neuroendocrine (*n* = 1), and salivary gland (*n* = 1).

**Table 2:** Radiosurgical and survival outcomes in the study cohort (*n* = 100).

Characteristic	Quantity
Median tumor volume, cm^3^	22.0 (14.1–69.5)
Median marginal dose, Gy	35.2 (24.9–41.5)
Local control rate at final follow-up	74 (74%)
Best response time after GKRS	
Median interval from GKRS, months	4 (1–36)
<12 months	86 (86%)
Maximum volume reduction after GKRS	
Median volume decrease, %	80 (22–100)
Volume decrease >95%	30 (30%)
Cumulative local control	
Mean, months	47.8 (40.2–55.5) *
1-year rate, %	72.7 (62.3–83.1) *
2-year rate, %	65.3 (53.5–77.1) *
3-year rate, %	59.9 (44.9–74.9) *
Progression-free survival	
Median, months	7.5 (4.6–10.4) *
1-year rate, %	37.2 (26.6–47.8) *
2-year rate, %	17.9 (8.7–27.1) *
3-year rate, %	14.0 (5.4–22.6) *
Overall survival	
Median, months	16.3 (11.1–21.5) *
1-year rate, %	56.9 (46.9–66.9) *
2-year rate, %	37.6 (27.6–47.6) *
3-year rate, %	25.7 (16.1–35.3) *

Abbreviations: Gy = gray; GKRS = Gamma Knife radiosurgery; * 95% confidence interval.

**Table 3:** Comparison of present study outcomes with selected hypofractionated stereotactic radiosurgery series for large brain metastases.

Authors	No	Volume (cm^3^)	Fractions	Dose (Gy)	1yr-LTC (%)	1yr-OS (%)	RN (%)
Kim et al. [38]	36	18.3 (10.0–50.3)	3 (2–4)	24 (20–30)	90.0	66.7	2.7
Park et al. [39]	17	21.2 (11.0–38.1)	3 (3–5)	27 (21–40)	100.0	93.3	0
Samanci et al. [49]	76	6 (4.0–22.2)	3 (3–5)	27 (21–30)	96.0	63.6	0
Noda et al. [37]	78	7 (4.0–55.8)	10 (3–15)	35 (27–45)	72.0	76.7	6.4
Mishra et al. [5]	90	16.0 (10.1–42.3)	3 (3–5)	27 (24–27)	79.2	NA	6.5
Present study	100	22.0 (14.1–69.5)	5	35 (25–42)	74.0	56.9	9.0

Abbreviations: No = number of tumors; Gy = gray; mo = months; LTC = local tumor control; OS = overall survival; RN = radiation necrosis; NA = not available.

## Data Availability

The data that support the findings of this study are available from the corresponding author upon reasonable request.

## Abbreviations

The following abbreviations are used in this manuscript:

GKRS	Gamma Knife radiosurgery
LTC	Local tumor control
PFS	Progression-free survival
OS	Overall survival
RN	Radiation necrosis
KPS	Karnofsky Performance Status
WBRT	Whole brain radiotherapy
